# Temporal and Spatial Profiling of Root Growth Revealed Novel Response of Maize Roots under Various Nitrogen Supplies in the Field

**DOI:** 10.1371/journal.pone.0037726

**Published:** 2012-05-18

**Authors:** Yunfeng Peng, Xuexian Li, Chunjian Li

**Affiliations:** Key Laboratory of Plant-Soil Interactions, Ministry of Education, Department of Plant Nutrition, China Agricultural University, Beijing, China; University of Illinois, United States of America

## Abstract

A challenge for Chinese agriculture is to limit the overapplication of nitrogen (N) without reducing grain yield. Roots take up N and participate in N assimilation, facilitating dry matter accumulation in grains. However, little is known about how the root system in soil profile responds to various N supplies. In the present study, N uptake, temporal and spatial distributions of maize roots, and soil mineral N (N_min_) were thoroughly studied under field conditions in three consecutive years. The results showed that in spite of transient stimulation of growth of early initiated nodal roots, N deficiency completely suppressed growth of the later-initiated nodal roots and accelerated root death, causing an early decrease in the total root length at the rapid vegetative growth stage of maize plants. Early N excess, deficiency, or delayed N topdressing reduced plant N content, resulting in a significant decrease in dry matter accumulation and grain yield. Notably, N overapplication led to N leaching that stimulated root growth in the 40–50 cm soil layer. It was concluded that the temporal and spatial growth patterns of maize roots were controlled by shoot growth and local soil N_min_, respectively. Improving N management involves not only controlling the total amount of chemical N fertilizer applied, but also synchronizing crop N demand and soil N supply by split N applications.

## Introduction

Doubling of the world food production over the past four decades is associated with a seven-fold increase in consumption of synthetic nitrogen (N) fertilizer in agricultural systems [1]. In China, a 71% increase in total annual grain production from 283 to 484 MT (million tons) from 1977 to 2005 was achieved at the cost of 271% increase in synthetic N fertilizer application (from 7.07 to 26.21 MT) over the same period [2]. Maize is one of three major cereal crops in China. Its average grain yield per hectare increased rapidly from 962 kg in 1949 to 5,166 kg in 2007 [3]. The consumption of synthetic N fertilizer in China increased rapidly during the same period, exceeding 32 MT in 2007, accounting for 30% of global N fertilizer production [4]. However, the average maize grain yield per hectare of 5166 kg was much lower than that in Western countries such as the USA, where it was 9359 kg in 2006 [5]. Although the high yield records are more than 15 Mg ha^−1^ in some experimental plots [6], [7], and even reached 21 Mg ha^−1^ in Shangdong Province in 2005 [8], this was obtained in small experimental plots and with high input costs. The amount of topdressing N fertilizer applied in the high-yield experimental plots varied from 450 to 720 kg N ha^−1^ [6], [8]. The continuous increase in fertilizer supply promotes yield increase on the one hand, and brings serious environmental problems on the other hand. Excessive N fertilization in intensive Chinese agricultural systems does not make significant contributions to grain yield but decreases nitrogen use efficiency (NUE), and increases the risk of N leaching to ground water and soil acidification [2], [9]–[11].

In addition to overapplication, N is often applied incorrectly in China. A survey of chemical N fertilizer application in five major maize-producing provinces in North China during 2001 and 2003 revealed that 31.2–78.3% of the farmers used only a single N application as base fertilizer before sowing [12]. A study of the effects of single N application as base fertilizer on spring maize yield in Jilin province with 110 field experiments in 2004 and 2005 indicated that single N application significantly reduced maize yield compared with optimized N management based on soil mineral N (N_min_) [13]. In maize 45–65% of the grain N is from pre-existing N in the stover before silking. The remaining 35–55% of the grain N originates from post-silking N uptake [1]. Nitrogen stress at a critical stage may lead to irreversible yield loss. In a greenhouse experiment, Subedi and Ma [14] found that restriction of N supply from seeding to 8-leaf stage could cause an irreparable reduction in maize ear size and kernel yield; however, there was no yield reduction when N was restricted from silking, or 3 weeks after silking, to physiological maturity. Newly developed maize hybrids often show reduced rates of visible leaf senescence, which allows a longer duration of photosynthesis and has a positive effect on N uptake during the grain-filling period [15]–[18]. Whether N fertilizer application after silking is needed in order to meet the increased N demand of plants in the reproductive growth stage, and how split application of chemical N fertilizer influences root growth and N uptake by plants as well as N movement in the soil, are questions that require addressing.

Chemical N fertilizer applied in the soil is taken up by roots and then assimilated and used by plants. Better root growth and synchronized N supply throughout the crop growing season are beneficial for maximizing fertilizer uptake, optimizing grain yield, and reducing N losses. Many scientists are starting to see roots as central to their efforts to produce crops with a better yield, efforts that go beyond the Green Revolution [19]. However, less attention has been paid to the temporal and spatial dynamics of root growth in the soil profile and how root growth responds to various N supplies [1], [20], partially because roots are tangled underground and difficult to study [19]. Few studies have reported root growth plasticity of cereals under different N regimes [1], [20]. In a short-term experiment under controlled conditions, N deficiency stimulates root growth, while N oversupply inhibits root growth [21]. Localized nitrate application stimulates lateral root growth (the localized stimulatory effect) [21], [22]. Unfortunately, these unsystematic experiments under controlled conditions may not represent real situations in the field. It is interesting to know how roots perform in the soil profile with heterogeneous N distribution in time and space, and whether the responses of root growth to the above-mentioned N applications are repeated in long-term field experiments.

Successful N management requires better understanding of N uptake by roots and synchronized N supply throughout the crop growing season. We hypothesized that N deficiency stimulated early root growth but reduced grain yield. By contrast, N over-application inhibited early root growth and increased potential risk of N leaching without yield increase. Improving N management involved not only controlling the amount of applied N fertilizer, but also synchronizing plant demand and N applications for better root growth and high grain yield. To test this hypothesis and further dissect response strategies of maize roots to various N supplies in the field, comprehensive field studies in three consecutive years (2007–2009) were conducted in the present work to examine temporal and spatial distribution patterns of maize roots, plant N uptake, and N_min_ in the soil profile during the whole growth period, under different chemical N regimes, especially by split application of N fertilizer.

## Materials and Methods

### Experimental design

The field experiments were conducted in three consecutive years (2007–2009) in three adjacent experimental sites at the Shangzhuang Experimental Station of the China Agricultural University, Beijing. The soil type at the study site is a calcareous alluvial soil with a silt loam texture (FAO classification) typical of the region. The soil N_min_ and related chemical properties of the experimental soils are shown in Table S1. Maize hybrid DH 3719 (‘stay-green’ cultivar), a popular hybrid in North China, was used in the experiments and sown on 28 April 2007, 27 April 2008, and 27 April 2009, and harvested on 23 September 2007, 19 September 2008, and 21 September 2009. Flooding irrigation before sowing was used to keep the available soil water content above 75%. The amount of rainfall during the maize growing season in the three years was 428 mm, 608 mm, and 216 mm, respectively. In addition, 50 mm and 43 mm of irrigation were applied on 17 June 2007 and 2 July 2009, respectively. The monthly rainfall during the study period is shown in Table S2. Maize was overseeded (three seeds) with hand planters and the plots were thinned at the seedling stage to a stand of 100,000 plants ha^−1^. The seeds were sown in alternating 20-cm- and 50-cm-wide rows. The distance between plants was 28 cm in each row. A randomized complete block design with four replicates in each treatment in each year was used. The plot sizes were 56 m^2^ (5.6×10 m), 40 m^2^ (5×8 m), and 56 m^2^ (5.6×10 m) in 2007, 2008, and 2009, respectively.

### Fertilization and treatments

There were four (2007 and 2008) or three (2009) N treatments: 1) 0 N as control; no chemical N fertilizer was applied. 2) N topdressing at and after tasseling (TDAT), and 3) N topdressing before tasseling (TDBT). In order to determine the importance of timing of N topdressing, a treatment with delayed N topdressing at and after tasseling was set. In 2007, 175 kg N ha^−1^ as base fertilizer was applied in the TDAT and TDBT treatments, and total amount of N fertilization was 230 and 395 kg N ha^−1^ in TDAT and TDBT, respectively. According to the results of N accumulation in plants and soil N_min_ after the last harvest in 2007, 250 kg N ha^−1^ was set in TDAT and TDBT in 2008 and 2009, in which 60 kg N ha^−1^ was applied as base fertilizer. The remaining N was applied before tasseling (TDBT) at V8 (the eighth leaf emerged with ligule visible) and V12 (the twelfth leaf emerged), or at and after tasseling (TDAT) at VT (tasseling stage) and R2 (grain blister stage), respectively. 4) Traditional N practice (450 N); according to numerous high-yield studies in China, the application rate in the traditional N practice was set at 450 kg N ha^−1^, in which 175 kg N ha^−1^ was applied as base fertilizer, 50, 170, and 55 kg N ha^−1^ in 2007 and 2008, and 120, 70, 85 kg N ha^−1^ in 2009 were applied in wide interrows by hand as topdressings at the V8, V12 and VT, respectively. Detailed rates and times of N application are shown in Table S3.

The rate and timing of phosphorus and potassium fertilization in each year were the same. In addition, zinc (Zn) was applied in each year as base fertilizer because of the slight Zn deficiency in the experimental region. A total of 135 kg ha^−1^ of P_2_O_5_ as triple superphosphate [Ca(H_2_PO_4_)_2_·H_2_O], 120 kg ha^−1^ of K_2_O as potassium sulfate [K_2_SO_4_], and 30 kg ha^−1^ of ZnSO_4_·7H_2_O were applied. Before sowing, 90 kg ha^−1^ P_2_O_5_, 80 kg ha^−1^ of K_2_O and 30 kg ha^−1^ of ZnSO_4_·7H_2_O were broadcasted and incorporated into the upper 0–15 cm of the soil by rotary tillage. Another 45 kg ha^−1^ of P_2_O_5_ at V12 and 40 kg ha^−1^ of K_2_O at VT were applied in wide interrows by hand as topdressings. Each topdressing (NPK) was applied after harvest.

#### Harvest 2007

Plants were harvested at 38 (the eighth leaf emerged with ligule visible, V8), 57 (the twelfth leaf emerged, V12), and 74 (tasseling, VT) days after sowing (DAS) before fertilization and at 105 (grain blister stage, R2) and 147 (physiological maturity, R6, when 50% of the plants showed black layer formation in the grains from the mid-portion of the ears) DAS. At harvest, six consecutive plants (three plants each from two narrow rows) were cut at the stem base, chopped to a fine consistency, dried to a constant weight at 60°C and ground into powder to determine aboveground dry weight and N content. To estimate grain yield, ears in the central part of 21 m^2^ (2007 and 2009) or 14 m^2^ (2008) in each plot were hand-harvested at physiological maturity. Kernels from six randomly selected ears were harvested individually by hand, weighed and calculated to 15.5% moisture content. N content in each plant sample was analyzed by using a modified Kjeldahl digestion method [23]. Briefly, 0.3–0.4 g oven-dried plant tissue was digested with H_2_SO_4_ (98%)+H_2_O_2_ at 380°C for 3–4 h in a digestion tube. The digested solution was cooled to room temperature and added deionized water to 100 ml. An aliquot of 5 ml uniform solution was distilled and titrated with standardized 0.01 N sulphuric acid. The total N content was calculated from the concentration of standardized sulphuric acid. After shoot excision at each harvest, three whole root systems were excavated from each plot and washed free of soil with tap water. Two root systems were dried immediately after harvest and used to assess dry weight and N content, and the other root system was stored at −20°C for measuring root length, including embryonic and different whorls of shoot-borne roots [24]. At root harvest, each root system was excavated with a soil volume of 28 cm (14 cm on each side of the plant base in intrarow direction)×35 cm (10 cm in narrow interrow and 25 cm in wide interrow) and a depth of 40 cm. The area of 28 cm×35 cm was the soil surface occupied by each plant at the plant density of 100,000 plants ha^−1^. In addition, at each harvest, five 2-cm-diameter soil cores per plot were collected and mixed to measure soil N_min_ (auger method, [25], [26]). Samples were collected from the 0–90 cm soil layers (in 30 cm increments) in the interrow area. All fresh samples were crushed, sieved through a 3 mm sieve in the field, and extracted immediately after transfer to the laboratory with 0.01 mol L^−1^ CaCl_2_ solution and analyzed for soil N_min_ (NH_4_^+^-N+NO_3_^−^-N) by continuous flow analysis (TRAACS 2000, Bran and Luebbe, Norderstedt, Germany) [9], [10].

#### Harvest 2008

Plants were harvested on 53 (V8), 71 (V12), 86 (VT) and 111 (R2) DAS before fertilization and on 130 and 145 (R6) DAS. At each sampling date, shoot harvest and determination of dry weight and N content as well as final dry grain yield were performed as in 2007. In addition, two whole root systems were sampled from each plot at each harvest as in 2007 to determine root dry weight and N content. In order to study the temporal and spatial distribution of maize roots and soil N_min_ during the whole growth period, a different method from that in 2007 was used to obtain root and soil samples at each harvest after shoot excision. Soil samples of 28 cm (width)×35 cm (length)×50 cm (depth) with 10 cm increments in each plot under different treatments were collected. There were five soil blocks of 28 cm×35 cm×10 cm in each plot. All visible roots in each soil block were separated in the field by hand and placed in individual marked plastic bags. These roots were washed free of soil after transfer to the laboratory and then frozen at −20°C until root length analyses were performed [24]. After root harvest, the soil in each block was crushed by hand and sieved through a 3 mm sieve in the field. A representative sample of the mixed soil was placed in a marked plastic bag for N_min_ extraction and analysis as performed in 2007.

#### Harvest 2009

Plants were harvested on 33, 45 (V8), 61 (V12), 80 (VT), 110 (R2) and 147 (R6) DAS. The methods for shoot and root harvest, dry weight and N content determination, root and soil sampling, and soil N_min_ measurement were identical to those used in 2008. The only difference was that the soil was excavated to a depth of 60 cm.

### Statistical analysis

Data were analyzed using analysis of variance with the SAS package (SAS Institute, 1996). Differences between data in all tables were tested with PROC ANOVA. N treatments were treated as fixed effects and means of different N treatments were compared based on least significant difference (LSD) at the significance level of 0.05.

## Results

### Temporal and spatial distribution patterns of maize roots

**Figure 1:**
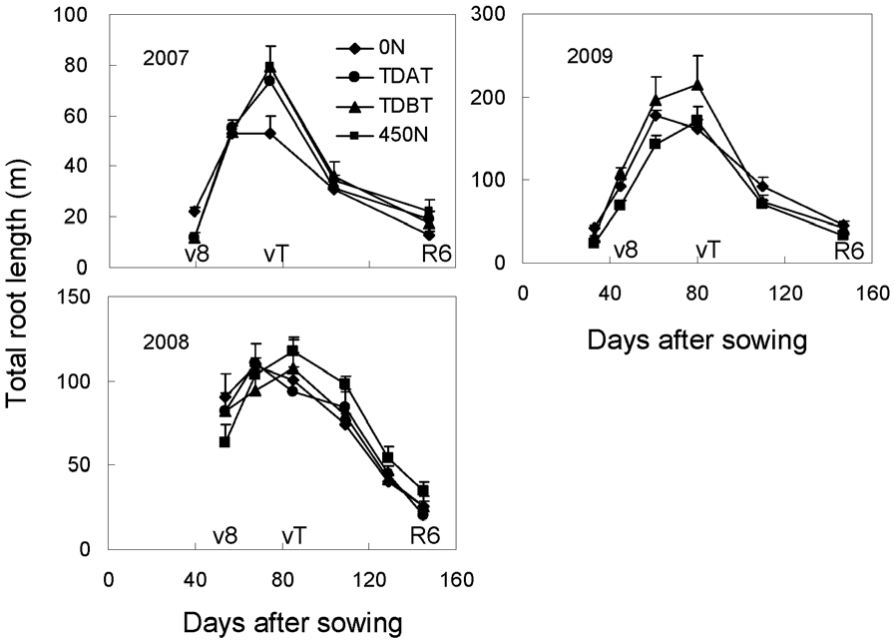
Total root length of maize plants during the whole growth period in response to N fertilization in three consecutive years. In 2007, the whole root system was excavated with a soil volume of 28 cm×35 cm and a depth of 40 cm. In 2008 and 2009, root systems were excavated within a soil volume of 28 cm×35 cm and a depth of 50 cm (2008) or 60 cm (2009) with 10 cm increments. The bars represent the standard error of the mean, *n* = 4. TDAT means N top dressing after tasseling. The total amount of N applied in TDAT treatment was 230 and 250 kg ha^−1^ in 2007 and 2008, respectively. TDBT means N top dressing before tasseling. The total amount of N applied in TDBT treatment was 395, 250 and 250 kg ha^−1^ in 2007, 2008 and 2009, respectively, and is the same in the following figures.

The total root length of maize plants increased dramatically after the V8 stage, peaked at the VT stage, and then declined rapidly until the R6 stage. The dynamic pattern of total root length over the entire growth period was consistent in all three years, irrespective of N regimes (Fig. 1). However, the total root length in the early growth stage was differentially regulated by base N treatments. N Deficiency (0 N) stimulated root growth in the early growth stage (V8 stage), and the total root length peaked before the VT stage, followed by an early decline compared to other treatments with base N fertilizer and N topdressing before tasseling in all three years. Similarly, the treatment with base N fertilizer and delayed N topdressing in 2008 also caused an early decrease in the total root length. In contrast, 175 kg ha^−1^ base N fertilizer (450 N treatment) inhibited root growth in the early growth period. The total root length in the following growth stages under 450 N treatment was comparable with that under TDBT treatment (with 60 kg base N fertilizer) in 2008 and 2009 (Fig. 1).

**Figure 2 pone-0037726-g002:**
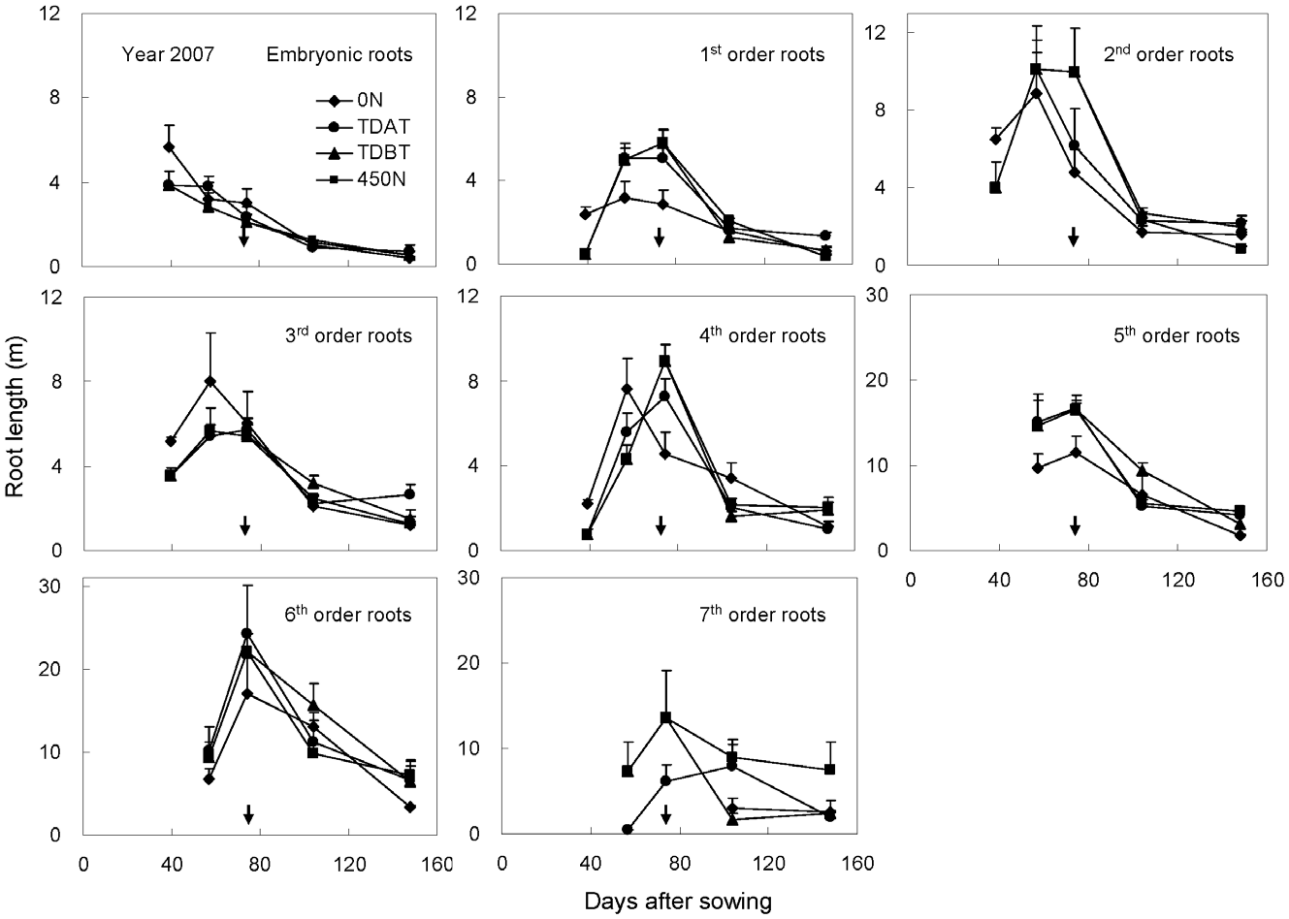
Length of embryonic roots and different whorls (1^st^ to 7^th^ orders) of nodal roots of maize plants in response to N fertilization in 2007. Arrows indicate the time of tasseling. Whole root systems were excavated with a soil volume of 28 cm×35 cm and a depth of 40 cm, and then separated into embryonic roots and different whorls of nodal roots. The bars represent the standard error of the mean, *n* = 4.

To further analyze dynamic changes in root structure under different N treatments, the total length of the embryonic roots and each whorl of nodal roots was monitored in 2007 by whole root excavation with a soil volume of 35 cm (length)×28 cm (width)×40 cm (depth) at each growth stage (Fig. 2). The embryonic root began to shorten after the first harvest; therefore, the total root length was largely determined by nodal roots that initiated with root development before VT (except for the 7^th^ whorl of nodal roots of N-deficient plants). The total length of the embryonic root and most nodal roots peaked at the VT stage and then decreased simultaneously until maturity. Although N deficiency (0 N) enhanced embryonic root growth before V12 (57 DAS), it negatively regulated nodal root growth and mortality. In particular, initiation and growth of the 7^th^ whorl of nodal roots of N-deficient plants after the VT stage was almost completely suppressed (Fig. 2).

**Figure 3 pone-0037726-g003:**
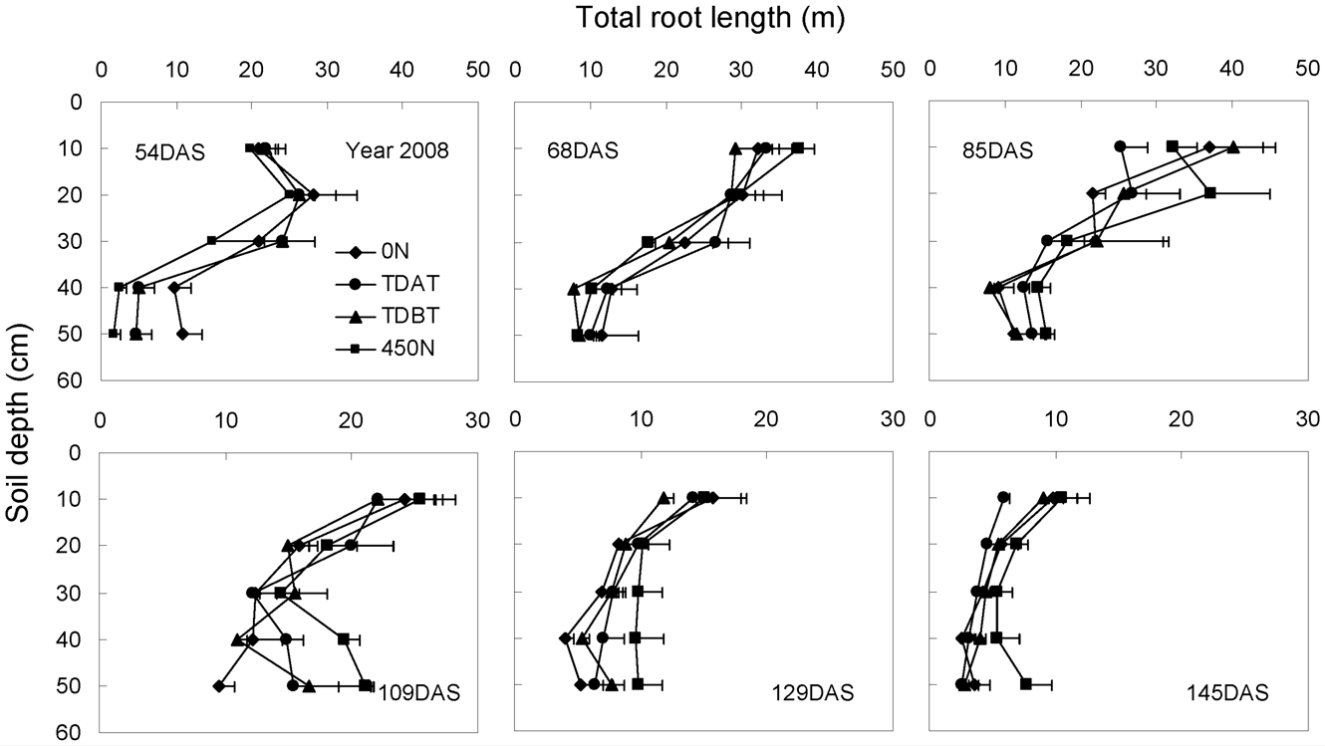
Total root length of maize plants in each soil layer at different growth stages in response to N fertilization in 2008. Root systems were excavated within a soil volume of 28 cm×35 cm and a depth of 50 cm with 10 cm increments. The bars represent the standard error of the mean, *n* = 4.

**Figure 4:**
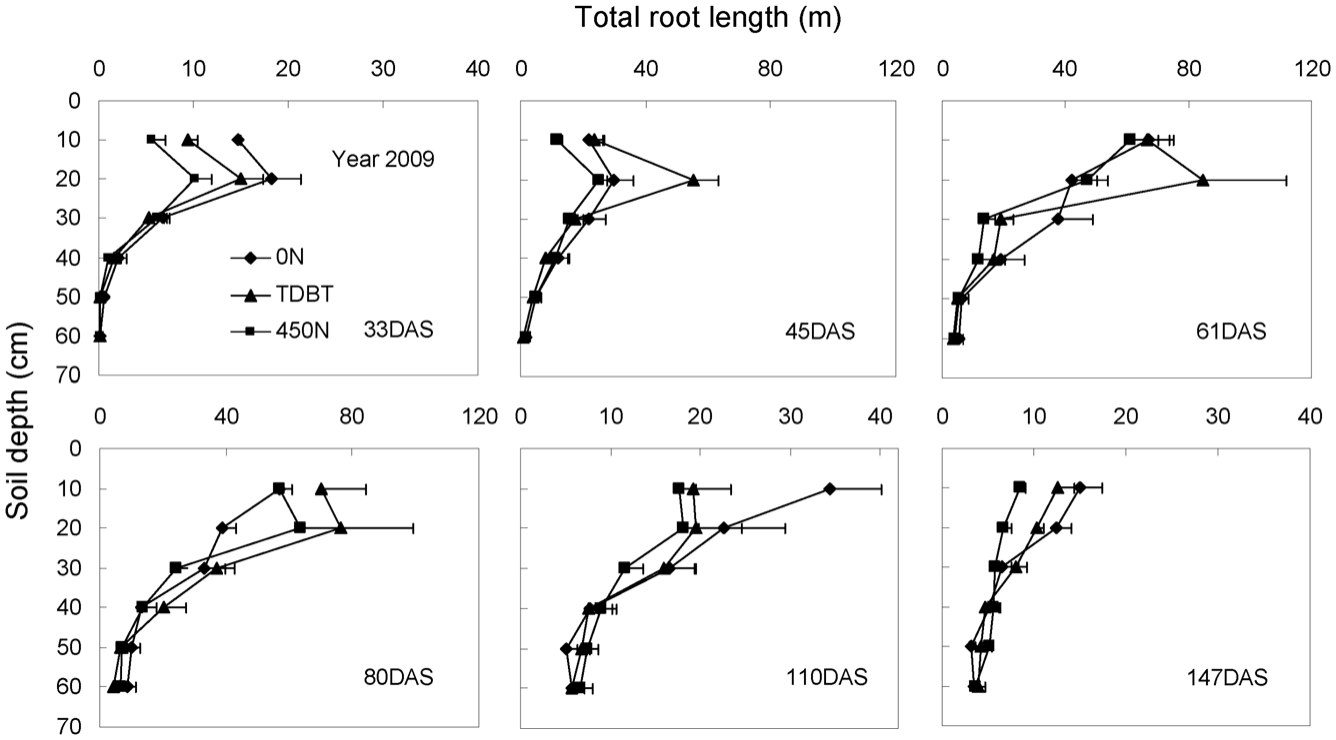
Total root length of maize plants in each soil layer at different growth stages in response to N fertilization in 2009. Roots were excavated within a soil volume of 28 cm×35 cm and a depth of 60 cm with 10 cm increments. The bars represent the standard error of the mean, *n* = 4.

Monitoring of temporal and spatial root distribution in 2008 and 2009 (Figs. 3, 4) with a different method also confirmed that N deficiency stimulated, while overapplication of base N fertilizer (450 N) inhibited, root growth in the early growth stage. More roots were distributed in upper soil layers than in deep soil layers in all growth stages. Moderate N input promoted root proliferation in the nutrient-rich soil profile during the fast root growth period in 2009 (V8-tasseling; Fig. 4). Maize roots had already grown into the 50–60 cm soil layer one month after sowing in 2009, regardless of N treatment. Interestingly, deep root growth was also stimulated at the reproductive growth stage in 2008, when N was overapplied (Fig. 3).

### Dry matter and N accumulation in the shoot and grain yield

The changes in shoot biomass and N accumulation were represented by a typical ‘S curve’. However, the increase in shoot biomass and N content was not synchronized. The rapid increase in shoot biomass began at the V8 stage and peaked during the V12–R2 stages, while the N content increased during sowing-V8 and peaked during the V8–VT stages (Tables 1, 2). The shoot biomass and N accumulation patterns were the same in all three years regardless of N regimes. The study site is located on the North China Plain. In this region, total environmental N inputs (atmospheric and irrigation contributions) reach about 104 kg ha^−1^ year^−1^. Ammonia volatilization and nitrate leaching are the main N loss pathways [2]. Based on N accumulation (Table 2) and soil N_min_ (see below) after the final harvest in 2007, total environmental N inputs in the growing season, and amount of N loss in the region, 250 kg N ha^−1^ fertilizer was applied in delayed N topdressing (TDAT) and moderate N (TDBT) treatments in 2008 and 2009. N deficiency (0 N) significantly reduced shoot biomass, N content, and grain yield; while N overapplication (450 N) failed to increase N uptake, shoot biomass, as well as grain yield, compared with TDBT in all three years. The timing of N topdressing affected shoot growth and N accumulation. Delayed N topdressing decreased N uptake and biomass accumulation during the V8–V12 period, and caused a decrease in final dry matter and grain yield, compared with the TDBT treatment in 2008.

**Table 1:** Shoot dry matter accumulation (t/ha) in different growth periods, final shoot dry weight (DW) and grain yield (t/ha) of maize plants supplied with different N rates in three years.

Year	Treatments	Growth period					Total DW	Grain yield
		Sowing-V8	V8-V12	V12-VT	VT-R2	R2-R6		
2007	0 N	0.9a	3.8c	3.9b	10.1b	−0.1a	18.6b	9.7b
	TDAT*	0.7a	4.8b	5.0b	13.9a	3.4a	27.8a	12.8a
	TDBT**	0.7a	5.5a	7.3a	8.5b	6.7a	28.7a	12.4a
	450 N	0.7a	5.5a	7.3a	9.2b	6.4a	29.0a	13.3a
2008	0 N	1.2b	2.9b	4.8a	6.8b	4.9b	20.6b	11.0c
	TDAT	1.6a	2.6b	5.2a	8.4ab	5.3ab	23.2ab	12.1b
	TDBT	1.6a	3.8a	4.3a	10.5a	7.0ab	27.2a	13.8a
	450 N	1.4ab	3.5a	4.2a	7.9ab	8.6a	25.6a	13.1a
2009	0 N	1.0a	2.2b	3.6b	4.7b	3.4a	14.9b	6.3b
	TDBT	1.1a	2.9a	5.4a	9.2a	2.0a	20.6a	10.7a
	450 N	1.1a	3.5a	5.4a	9.7a	1.5a	21.2a	11.0a

Values in columns in each year followed by a different letter represent a significant difference between N treatments (*P*<0.05). Values are means ± SE (*n* = 4).

* TDAT, N topdressing after tasseling. The total amount of N applied in the TDAT treatment was 230 and 250 kg/ha in 2007 and 2008, respectively, and is the same in the following tables.

** TDBT, N topdressing before tasseling. The total amount of N applied in the TDBT treatment was 395, 250 and 250 kg/ha in 2007, 2008 and 2009, respectively, and is the same in the following tables.

V8, the eighth leaf emerged with ligule visible; V12, the twelfth leaf emerged; VT, tasseling; R2, grain blister stage; R6, physiological maturity, and they are the same in the following table.

**Table 2:** Shoot N accumulation (kg/ha) in different growth periods and the final shoot N content of maize plants supplied with different N rates in three years.

Year	Treatments	Growth period					Total N content
		Sowing-V8	V8-V12	V12-VT	VT-R2	R2-R6	
2007	0 N	22a	25c	53b	32ab	−8b	124b
	TDAT	22a	123a	35b	86a	5ab	271a
	TDBT	22a	111b	97a	11b	27ab	267a
	450 N	22a	111b	97a	25b	27a	282a
2008	0 N	36b	35b	37a	41a	27a	176b
	TDAT	52a	33b	53a	52a	26a	218ab
	TDBT	52a	64a	49a	61a	46a	273a
	450 N	44ab	73a	47a	35a	55a	254a
2009	0 N	19a	23c	18b	25a	24a	109b
	TDBT	25a	46b	67a	53a	11a	202a
	450 N	30a	58a	72a	46a	5a	210a

Values in columns in each year followed by a different letter represent a significant difference between N treatments (*P*<0.05). Values are means ± SE (*n* = 4).

### Temporal and spatial dynamics of soil N_min_

**Figure 5:**
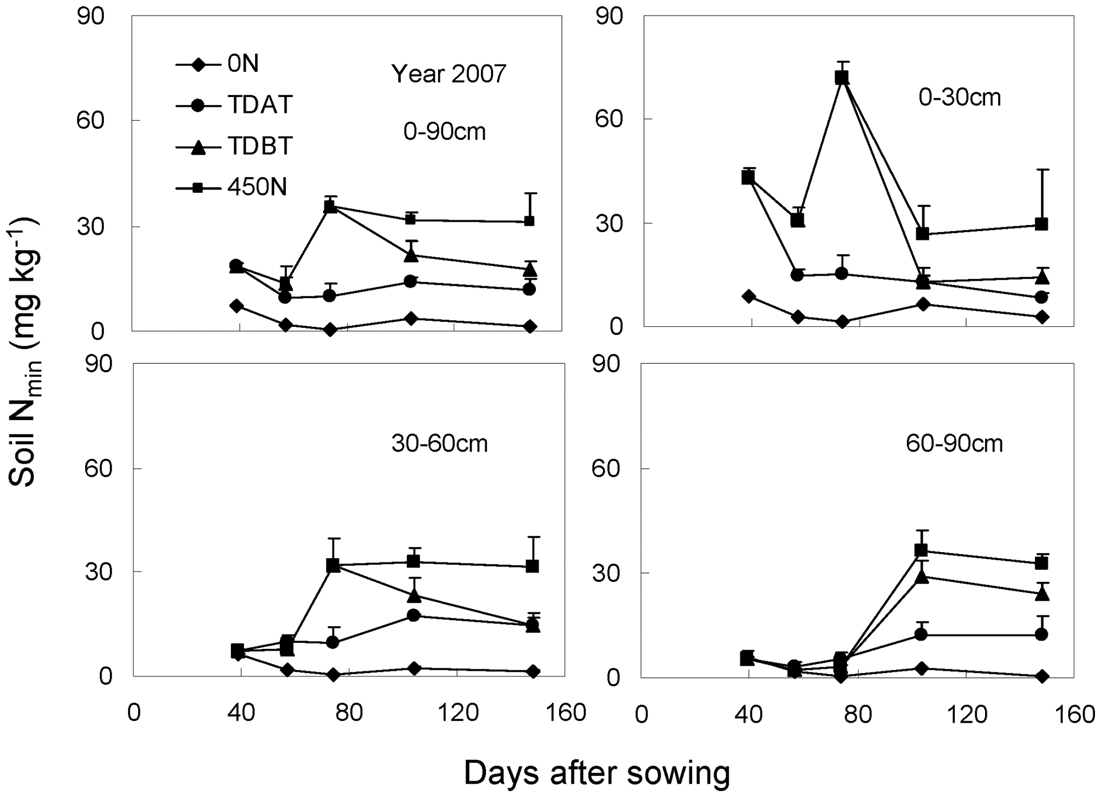
Soil mineral nitrogen (N_min_; NH_4_^+^-N+NO_3_^−^-N) in the 0–90 cm soil profile in response to N fertilization in 2007. The soil samples were obtained using the soil auger method at the same time in each plant harvest. The bars represent the standard error of the mean, *n* = 4.

**Figure 6 pone-0037726-g006:**
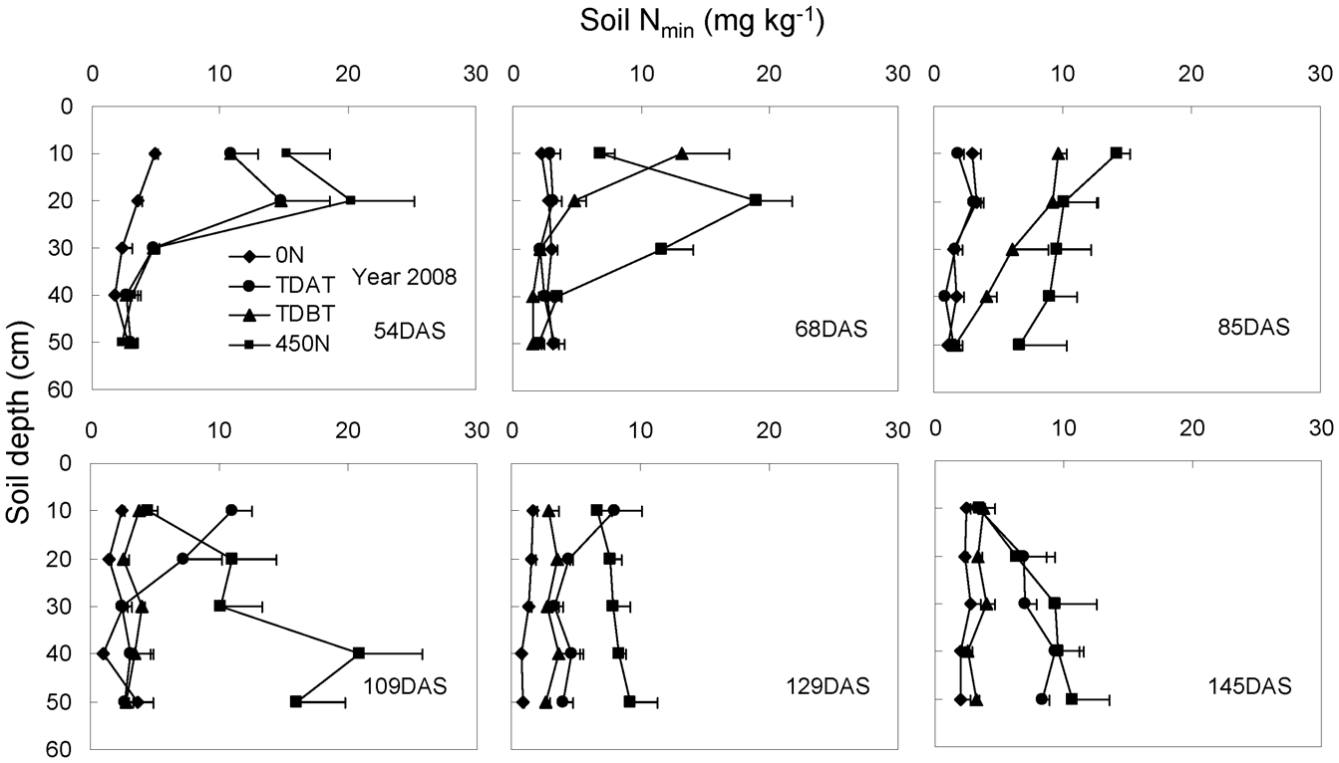
Temporal and spatial distribution of soil mineral nitrogen (N_min_; NH_4_^+^-N+NO_3_^−^-N) in the 0–50 cm soil profile in response to N fertilization in 2008. The soil samples were obtained by excavating soil layers within a soil volume of 28 cm×35 cm and a total depth of 50 cm with 10 cm increments at each time after shoot harvest. The bars represent the standard error of the mean, *n* = 4.

In 2007, 0–90 cm soil samples were collected in 30 cm increments in the interrow area at each harvest following the auger method. In 2008 and 2009, soil samples were obtained by digging soil blocks within a soil volume of 35 cm (length)×28 cm (width)×50 (or 60) cm (depth), with 10 cm increments at each time after shoot harvest. Irrespective of methods used for soil sample collection, our three-year experiment consistently demonstrated that the soil N_min_ in each soil layer at each growth stage was positively correlated with the amount of N application (Figs. 5–7). In the 0 N treatment, soil N_min_ was very low and remained relatively steady at each harvest in all three years, in spite of the continuous increase in plant N content with time (Table 2). In the 450 N treatment in all three years, however, high N accumulation in soil profiles was observed, even after the last harvest, indicative of N overapplication. This was also true for the TDBT treatment in 2007 when 395 kg N ha^−1^ was applied. N overapplication led to obvious temporal and spatial fluctuations in soil N_min_ and strong N leaching to the deeper soil layer occurred upon heavy rainfall in 2007 and 2008 growth seasons (Figs. 5, 6). Additionally, late N topdressing also caused N leaching to deep soil layers in 2008, although 250 kg N in total was applied (TDAT, Fig. 6). In comparison, moderate N application (TDBT) ensured plant demand without N leaching.

**Figure 7:**
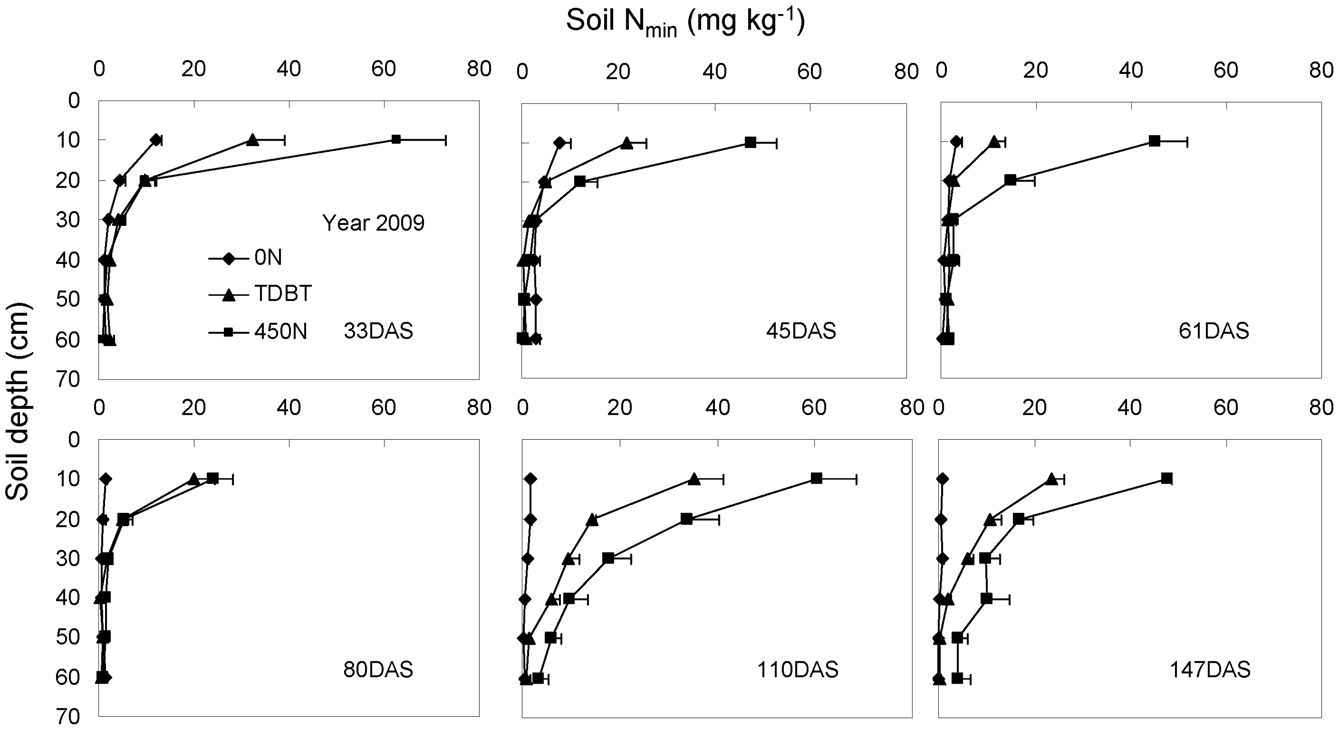
Temporal and spatial distribution of soil mineral nitrogen (N_min_; NH_4_^+^-N+NO_3_^−^-N) in the 0–60 cm soil profile in response to N fertilization in 2009. The soil samples were obtained by excavating soil layers within a soil volume of 28 cm×35 cm and a total depth of 60 cm with 10 cm increments at each time after shoot harvest. The bars represent the standard error of the mean, *n* = 4.

## Discussion

### Influence on maize root growth

The root system plays predominant roles in nutrient uptake for plant growth and yield formation [24]. Previous studies indicated that optimized N application is beneficial for maize shoot growth and grain yield [2], [10], [27], [28]. However, little is known about how root development responds to different N supply in the soil profile. The present work showed that N deficiency stimulated early root growth as indicated by increased length of early-initiated nodal roots. This stimulatory effect lasted only for a short time and the total root length began to decrease when maize plants were still in the rapid vegetative growth stage (Fig. 1) with high N uptake activity (Table 2). The early decline in total root length under N deficiency was due to early mortality of the early-initiated nodal roots and growth suppression of the later-initiated nodal roots (Fig. 2). Root growth is closely associated with assimilate supply from the shoot [29]. The stimulated root growth in the early growth stage under N deficiency was achieved at the cost of reduced shoot growth [30], which led to insufficient carbon supply for continuous growth of early-initiated nodal roots and rapid elongation of later-initiated nodal roots. As a result, total plant N content was significantly reduced (Table 2). In contrast, the total root length of maize plants supplied with sufficient N didn't decrease until tasseling (Fig. 1), which favored robust nutrient uptake early in the growing season, and nutrient translocation from roots to reproductive organs later in the season [24], [31]–[34].

Overapplication of base N fertilizer (175 kg ha^−1^ in the 450 N treatment) inhibited early root growth of maize plants, compared with other treatments in all three years (Figs. 1–4). The results suggested the ‘systemic inhibitory effect’ of high external N concentration on root growth [21] applied in the field. It is envisaged that a single application of total N as base fertilizer in the five major maize producing provinces in North China [12] would more dramatically inhibit early root growth. A single application of total N before sowing reduced maize yield significantly compared with split applications of chemical N fertilizer based on the soil N_min_ test. However, this reduction of grain yield was not only because of the inhibited root growth in the early growth stage owing to N toxicity, but also because of N deficiency in the reproductive stage owing to N losses by different ways [2], [35].

Although maize rooting depth at anthesis varies from around 0.7 m to close to 1 m, approximately 90% of roots grow in the upper 0.3 m soil [36]. Consistently, the present results in 2008 and 2009 (Figs. 3, 4) showed that most of the root length was distributed in the upper 30 cm soil, regardless of N treatments. The decrease in the total root length after tasseling indicates rapid root death that is mainly attributed to lateral root mortality [24], especially in the upper 30 cm soil layer. The root length in deep soil layers (40–60 cm) was quite constant during the whole growth period. Notably, maize roots could sense the changes in soil N_min_. A localized stimulatory effect of nitrate-N patches on root growth has been reported when the whole root system suffered from N deficiency [21], [22]. In this process, nitrate serves as a signal, and the nitrate transporter CHL1 functions as a nitrate sensor [21], [37], [38]. The majority of the total root length of N-treated plants was distributed in the upper 30 cm soil layer before tasseling in 2008 and during the whole growth period in 2009, because of high soil N_min_ in this soil layer (Figs. 3, 4, 6, 7). However, there was an obvious increase in root length of maize plants supplied with 450 N in the 40–50 cm soil layer from 109 d after sowing to the last harvest in 2008 (Fig. 3). During the same period increased soil N_min_ owing to N leaching in the same soil profile was also observed (Fig. 6). Together, our study provides direct evidence that N-sufficient maize plants could respond to temporally and spatially heterogeneous soil N_min_ via enhanced root proliferation in the soil profile with higher N_min_.

Water stress significantly reduces maize N uptake, accelerates leaf senescence, and thus reduces grain yield, compared with the well-watered plants [39], [40]. This is supported by the results in the present study that shoot dry weight, N content and grain yield of all treated plants in 2009 were the lowest among the three years (Tables 1, 2), because of the limited precipitation in this year (Table S2). By contrast, the total root length of all treated maize plants in 2009 was extremely high (Fig. 1). Roots are less sensitive to water deficits than leaf, stem or silks growth [41], [42]. The results in the present study indicated that maize root growth could be stimulated by low water potential. On the other hand, soil water content influences nutrient availability [30]. Low soil water availability causes low N flux to the root surface. Enhanced root growth (Fig. 1) is beneficial for plants to capture more N under dry soil conditions.

### N application, uptake and grain yield

Under N deficiency, grain yield is negatively correlated with early root growth due to competition for N resources [1], [43]. In order to obtain high grain yield, fertilizer overapplication in Chinese intensive agricultural systems is very common, since farmers believe that additional fertilizers further improve crop yield [2]. In fact, N overapplication not only inhibited early root growth as discussed above, but also failed to increase shoot dry weight and grain yield of maize plants (Table 1; [44]). N overapplication did not increase total plant N content either, compared with the moderate N treatment (TDBT) in 2008 and 2009 (Table 2). The shoot N concentration of maize plants was the same under 450 N and TDBT treatments at each sampling time. Therefore, excessive N could not be taken up by plants and used to increase grain yield, but instead would increase the risk of N leaching and potential environmental pollution.

Besides quantity control, timing of fertilization is also important. The results in the present study indicated that the increases in shoot biomass and N content were not synchronized. Approximately 60–86% of the total N in maize plants (except the 0 N treatment in 2009) was taken up before tasseling, whereas 53–64% of the dry matter was accumulated after tasseling in all three years (Table 3). Therefore, N topdressing after the V8 stage (TDBT) was necessary to ensure adequate soil N supply for rapid plant growth. Delayed N topdressing (TDAT) decreased not only plant N content and dry matter accumulation during the V8–V12 period, but also caused a decrease in final dry matter and grain yield, compared with the TDBT treatment in 2008 (Tables 1, 2). It is reported that N application rate influences production of maize spikelets and the size of the developing ears before flowering [45]. The period from 1–2 weeks before to 2 weeks after silking is critical for establishment of a large grain sink of maize plants [46]. TDAT could not compensate for the reduction in plant N content and shoot growth caused by insufficient N supply in the vegetative growth stage, although total N supply was sufficient. Moreover, because of the rapid mortality of the maize root system (Fig. 1) and the slower plant N content increase (Table 3) after tasseling, remaining N fertilizer and mineralized N from the soil were sufficient to meet the plant demand during the reproductive growth stage. Additional unnecessary N application during this stage would increase the risk of N losses. The results indicate that even in intensive agricultural systems with ‘stay-green’ maize cultivars, N topdressing should be applied before tasseling to maximally synchronize with crop N demand.

**Table 3:** Ratio of DW and N uptake presilking and total accumulation in maize plants with different N treatments in 2007, 2008 and 2009.

Year	Treatments	Ratio DW pre silking/total accumulation	Ratio N uptake pre silking/total accumulation
2007	0 N	0.46a	0.81a
	TDAT	0.38a	0.66a
	TDBT	0.47a	0.86a
	450 N	0.47a	0.82a
2008	0 N	0.43a	0.61a
	TDAT	0.41ab	0.63a
	TDBT	0.36b	0.60a
	450 N	0.36b	0.65a
2009	0 N	0.46a	0.55b
	TDAT	0.46a	0.68ab
	450 N	0.47a	0.76a

Values in columns represent the significant differences between N treatments, (*P*<0.05). Means ± SE, *n* = 4.

### Influence on soil N_min_

In the 0 N treatment in all three years, soil N_min_ values were very low and remained almost constant in the studied soil profile during the whole maize growth period, except in the 0–10 cm soil layer at the elongation stage (Figs. 5–7). The continuous increase in shoot N content of the 0 N-treated maize plants indicated rapid soil N mineralization in the soil surrounding roots. Studies using the annual grass *Avena barbata* show that the rate of gross N mineralization in rhizosphere soil is about 10-fold higher than that in bulk soil [47]. By contrast, overapplication of chemical N fertilizer in all three years caused obvious N fixation (adsorption in soil lattice and transformation into organic N by soil microorganism) and accumulation in the soil. The more base N fertilizer was applied, the more N was fixed in the soil (Figs. 5–7). High N accumulation in soil profiles not only decreases NUE, but also causes environmental pollution in intensive agricultural systems [48], [49]. N leaching is closely correlated to precipitation and soil moisture. With heavy summer rainfall in 2007 and 2008, soil N_min_ moved downward, resulting in high nitrate accumulation in deep soil profiles (Figs. 5, 6; [9]). Even delayed N topdressing in 2008 (TDAT, moderated N input) caused N leaching to deep soil layers (Fig. 6). In comparison with overapplication of chemical N fertilizer, soil N_min_ in the root zone remained at a relatively low level under the TDBT treatment, while maintaining grain yield (Table 1).

### Conclusions

With sufficient or very frequently excessive N supply, the highest maize yield reached in North China in recent years was 21 Mg ha^−1^ [8]. The root system has played essential roles in N uptake and dry matter transformation in improving maize grain yield. However, it remained unclear how the root system responded to current fertilization regimes. The present study showed that vast majority of roots grow in the upper 30 cm soil layer. The root length in deep soil layers (40–60 cm) was quite constant during the whole growth period. The decrease in the total root length after tasseling was mainly due to rapid lateral root death in the top 30 cm soil layer. N deficiency stimulated early initiated nodal root growth only for a short time; however, it completely suppressed initiation and growth of the 7^th^ whorl of nodal roots and accelerated older nodal root death, causing an early decrease in the total root length when maize plants were still in the rapid vegetative growth stage with high N uptake activity. In contrast, 175 kg ha^−1^ base N fertilizer (450 N treatment) inhibited root growth in the early growth period. Importantly, root length increased in the 40–50 cm soil layer in response to N leaching, even under sufficient N supply (450 N) in 2008, indicating that N-sufficient maize plants responded to local N resources via enhanced root proliferation in the soil profile with higher N_min_. Further, N uptake and accumulation primarily occurred before tasseling, prior to substantial dry matter accumulation and grain formation. Therefore, appropriate N topdressing before tasseling (after the V8 stage) was necessary to ensure adequate N supply for robust plant growth and development. Early N excess, deficiency or delayed N topdressing reduced total N uptake, resulting in a significant decrease in dry matter accumulation and grain yield. Lastly, the soil in the field has great buffering capacity to maintain relatively constant N_min_ in most soil profiles while supporting maize growth under the 0 N treatment in all three years. N overapplication or delayed N topdressing caused N accumulation in the soil and leaching towards deep soil profiles and groundwater upon heavy rainfall in the growth season. TDBT treatment appears to be a good N application strategy because it maintains superior root growth relative to other N application strategies during the maize growing period, and significantly reduces N loss without sacrificing grain yield.

## Supporting Information

Table S1 Total soil mineral nitrogen and selected soil chemical properties before maize planting in 2007, 2008 and 2009.
(DOCX)

Table S2 Monthly rainfall during the maize growing period in 2007, 2008 and 2009.
(DOCX)

Table S3 Rates and times of chemical N application in the field experiments in 2007, 2008 and 2009.
(DOCX)
