# Supplementary material for: Temporal and Spatial Profiling of Root Growth Revealed Novel Response of Maize Roots under Various Nitrogen Supplies in the Field
Source: PLoS One. 2012 May 18;7(5):e37726. doi: 10.1371/journal.pone.0037726 (PMC3356300; doi:10.1371/journal.pone.0037726)
Supplement: Table S1 — Total soil mineral nitrogen and selected soil chemical properties before maize planting in 2007, 2008 and 2009. (DOCX) [file pone.0037726.s001.docx]

Table S1. Total soil mineral nitrogen (N_min_; NH_4_^+^ + NO_3_^−^ in the 0–90 cm soil layer) and selected soil chemical properties (in the 0–30 cm soil layer) before maize planting in 2007, 2008 and 2009. The soil was sampled by the auger method in each year

| Year | Soil N_min_ (kg/ha) | pH | O.M.(g/kg) | Total N (g/kg) | Olsen-P (mg/kg) | NH_4_OAc-K (mg/kg) |
| --- | --- | --- | --- | --- | --- | --- |
| 2007 | 62 | 8.00 | 8.40 | 0.74 | 10.16 | 78.00 |
| 2008 | 62 | 8.00 | 11.50 | 0.83 | 7.63 | 76.30 |
| 2009 | 60 | 7.86 | 7.27 | 0.71 | 7.14 | 97.59 |
