# Supplementary material for: Temporal and Spatial Profiling of Root Growth Revealed Novel Response of Maize Roots under Various Nitrogen Supplies in the Field
Source: PLoS One. 2012 May 18;7(5):e37726. doi: 10.1371/journal.pone.0037726 (PMC3356300; doi:10.1371/journal.pone.0037726)
Supplement: Table S2 — Monthly rainfall during the maize growing period in 2007, 2008 and 2009. (DOCX) [file pone.0037726.s002.docx]

Table S2. Monthly rainfall during the maize growing period in 2007, 2008 and 2009

| Year | May | June | July | August | September | Total rainfall (mm) |
| --- | --- | --- | --- | --- | --- | --- |
| 2007 | 54 | 75 | 183 | 59 | 57 | 428 |
| 2008 | 56 | 112 | 157 | 213 | 71 | 608 |
| 2009 | 11 | 39 | 75 | 27 | 65 | 216 |
