# Supplementary material for: Temporal and Spatial Profiling of Root Growth Revealed Novel Response of Maize Roots under Various Nitrogen Supplies in the Field
Source: PLoS One. 2012 May 18;7(5):e37726. doi: 10.1371/journal.pone.0037726 (PMC3356300; doi:10.1371/journal.pone.0037726)
Supplement: Table S3 — Rates and times of chemical N application in the field experiments in 2007, 2008 and 2009. (DOCX) [file pone.0037726.s003.docx]

Table S3. Rates and times of chemical N application in the field experiments in 2007, 2008 and 2009.

| Year | Treatments | Before sowing | V6 | V10 | VT | R2 | Total N (kg/ha) |
| --- | --- | --- | --- | --- | --- | --- | --- |
| 2007 | 0N |  |  |  |  |  | 0 |
|  | TDAT* | 175 |  |  | 55 |  | 230 |
|  | TDBT** | 175 | 50 | 170 |  |  | 395 |
|  | 450N | 175 | 50 | 170 | 55 |  | 450 |
| 2008 | 0N |  |  |  |  |  | 0 |
|  | TDAT | 60 |  |  | 120 | 70 | 250 |
|  | TDBT | 60 | 120 | 70 |  |  | 250 |
|  | 450N | 175 | 50 | 170 | 55 |  | 450 |
| 2009 | 0N |  |  |  |  |  | 0 |
|  | TDBT | 60 | 120 | 70 |  |  | 250 |
|  | 450N | 175 | 120 | 70 | 85 |  | 450 |

* TDAT means N top dressing after tasseling. The total amount of N applied in TDAT treatment was 230 and 250 kg ha^-1^ in 2007 and 2008, respectively (same in the following table).

** TDBT means N top dressing before tasseling. The total amount of N applied in TDBT treatment was 395, 250 and 250 kg ha^-1^ in 2007, 2008 and 2009, respectively (same in the following table).
